# Apatinib for patients with advanced or recurrent cervical cancer: study protocol for an open-label randomized controlled trial

**DOI:** 10.1186/s13063-018-2858-2

**Published:** 2018-09-17

**Authors:** Jian-Guo Zhou, Nan-Jing Zhou, Qiong Zhang, Yao-yao Feng, Hang Zhou

**Affiliations:** Department of Oncology, Affiliated Hospital of Zunyi Medical University, NO.149, Dalian Road, Zunyi, 563000 China

**Keywords:** Apatinib, Advanced, Recurrent, Cervical cancer

## Abstract

**Background:**

In China, cervical cancer is the fifth most commonly diagnosed cancer, and the outcomes for patients with advanced or recurrent disease are poor. Apatinib, a small molecule inhibitor of vascular endothelial growth factor receptor (VEGFR-2), is an orally bioavailable agent, which has shown survival benefit in multiple solid tumors. Based on previous research, this phase II clinical trial aims to verify apatinib’s efficacy and safety in patients with advanced or recurrent cervical cancer.

**Methods/design:**

This randomized, parallel arm, open-label, interventional trial will be carried out to evaluate the efficacy and the safety of apatinib for advanced or recurrent cervical cancer. A total of 60 eligible patients will be allocated by intention, in a ratio of 1:1, to either the experimental group or the control group. The primary endpoint is progression-free survival, the secondary endpoints include overall survival, disease control rate, objective response rate, quality of life, and adverse events. Assessments will be carried out before enrolment (baseline) and every 4 weeks after treatment.

**Discussion:**

The aim of this trial is to demonstrate the clinical effect, safety, and side effects of apatinib in the treatment of advanced or recurrent cervical cancer. This study will clarify the efficacy and safety of this regimen.

**Trial registration:**

Chinese Clinical Trials Registry, ChiCTR-OIN-17012164. Registered on 24 July 2017.

**Electronic supplementary material:**

The online version of this article (10.1186/s13063-018-2858-2) contains supplementary material, which is available to authorized users.

## Background

Cervical cancer is the fourth most common malignancy diagnosed in women worldwide [1]. Although the incidence of cervical cancer has declined in recent years, in China, cervical cancer is the fifth most common cancer, and it has been estimated to account for 98,900 new cases and 30,900 deaths in 2015 [2]. Despite advances in cervical cancer treatment, outcomes for patients with advanced or recurrent disease are poor. Meanwhile, recurrent or advanced disease progression with metastasis is by far the most important reason for cancer-related deaths.

Since the late 1980s, several phase II trials have shown that single cisplatin has a higher response rate than other agents, for example, carboplatin and iproplatin [3–6]. Based on the advantages in clinical effect, toxicity, and feasibility, cisplatin became the standard therapy for the treatment of advanced or recurrent cervical cancer. In 2004, a randomized phase III study by Moore et al. demonstrated that paclitaxel plus cisplatin (TP) had better median progression-free survival (PFS; 4.8 months) and median overall survival (OS; 9.7 months) [7]. Moreover, the Gynecologic Oncology Group showed that the response rate, PFS, and OS are better for TP compared with vinorelbine plus cisplatin, gemcitabine plus cisplatin, and topotecan plus cisplatin [8]. Based on this research, the National Comprehensive Cancer Network guideline recommends TP as the standard regimen.

Progress in the understanding of the biological events underlying cancer development and progression has led to the design of molecular-targeted therapies for cancer, and several new compounds are presently under investigation in the clinical setting, such as the vascular endothelial growth factor (VEGF) inhibitor bevacizumab. In 2014, GOG240 demonstrated that there was a statistically significant improvement in PFS (8.2 vs 5.9 months) and OS (17 vs 13.3 months) with the addition of bevacizumab to chemotherapy [9]. On 14 August 2014, the Food and Drug Administration approved bevacizumab for patients with recurrent or advanced cervical cancer.

However, bevacizumab has been reported to lead to a higher rate of gastrointestinal perforations and recto-vaginal or vesico-vaginal fistulas, which are rare but severe. Furthermore, the occurrence of fistulas was observed in cervical cancer more frequently than other diseases treated with bevacizumab in combination [10]. In addition, bevacizumab can block angiogenesis by inhibiting vascular expansion directly and activating tissue factors. Considering these complications, we tried to find another agent with lower toxicity that was easier to administer and had a more acceptable price while still having the same efficacy compared with bevacizumab.

In recent years, targeted therapies have shifted the traditional treatment mode of cancers. Since 2010, several trials have indicated that apatinib, also known as YN968D1, has a clinical benefit across a broad range of malignancies, including gastric cancer, breast cancer, non-small-cell lung cancer, and hepatocellular carcinoma [11–15]. Apatinib is a tyrosine kinase inhibitor that selectively inhibits the vascular endothelial growth factor receptor-2 (VEGFR-2), which could inhibit VEGF-stimulated endothelial cell migration and proliferation, decrease tumor microvascular density, and block the formation of new blood vessels in tumor tissue [16]. Recently, a study by Xie et al. using apatinib for cervical cancer showed a survival benefit regarding the median PFS (8 months) and objective response rate (46.2%) [17]. However, this was a retrospective study, and we have designed this prospective study to evaluate the efficacy of apatinib in the treatment of advanced recurrent cervical cancer.

## Methods/design

### Study design

This study is designed as an open-label phase II single-center trial to demonstrate the non-inferiority of apatinib compared with standard TP using PFS as the primary endpoint.

### Inclusion criteria

The inclusion criteria are as follows:

(i) histologically confirmed uterine cervical cancer
(ii) at least one measurable lesion defined according to the Response Evaluation Criteria in Solid Tumors (RECIST 1.1)
(iii) one prior systemic chemotherapy regimen (paclitaxel plus cisplatin, topotecan plus cisplatin, bevacizumab, and so on)
(iv) recovered from the effects of any prior therapy (at least 4 weeks from the last surgery or the administration of chemotherapy)
(v) age ≥18 years
(vi) Eastern Cooperative Oncology Group performance status of 0–2
(vii) voluntarily agreed to participate by giving written informed consent for the trial
(viii) women with childbearing potential will be required to have a negative urine or serum pregnancy test within 24h prior to receiving the first dose of study medication and women should not be breastfeeding
(ix) adequate organ function:

- hemoglobin >8.0 g/dL
- neutrophils >2000 cells/μL
- platelets >75,000 cells/μL
- serum total bilirubin ≤1.5 mg/dL
- Aspartate transaminase (or serum glutamic oxaloacetic transaminase) and alanine transaminase (or serum glutamate-pyruvate transaminase) ≤2.5 × upper limit of normal (ULN) or ≤5 × ULN for liver metastasis
- serum creatinine ≤1.2 mg/dL and creatinine clearance ≥60 mL/min
- international normalized ratio <1.5 and activated partial thromboplastin time <1.5 × ULN
- serum albumin ≥30 g/L

### Exclusion criteria

Participants are excluded if they meet any of the following criteria:

(i) are not willing to sign the consent form
(ii) have a history or current evidence of any condition, therapy, or laboratory abnormality that might confound the results of the study
(iii) are currently participating in and receiving study therapy from another clinical trial
(iv) have participated in a study of an investigational agent or have used an investigational device within 4 weeks prior to the first dose of study treatment
(v) have legal incapacity or limited legal capacity or are receiving other oncology-specific medications not authorized in the protocol
(vi) have hypersensitivity to apatinib or its excipients or have poorly controlled hypertension (persisting for more than 7 days after antihypertensive treatment)
(vii) have unstable angina
(viii) are unable to self-administer oral medication
(ix) have impaired immune function, abnormal coagulation, or proteinuria
(x) have radiographically confirmed invasion of blood vessels, or abdominal fistula, gastrointestinal perforations, or abdominal abscess in the prior 6 months

In addition, participants with a mental health illness or mental symptoms that could affect their decision to participate or who are unable to take part in this study satisfactorily (as judged by the researchers) will also be excluded.

### Specific aims

This prospective study aims to evaluate the clinical benefits and side effects of apatinib for patients with advanced recurrent cervical cancer. The primary endpoint of this study is PFS, which is the time from randomization until death or progression, whichever comes first. Patients alive and free of progression at the last follow-up for progression were censored for PFS on the date of last follow-up.

The secondary endpoints include:

- OS, which is defined as the time from transplant until death; patients alive at last contact are censored for OS on the date of last contact
- disease control rate, which is defined as the proportion of patients who had a best response rating of complete response, partial response, or stable disease
- objective response rate, which is defined as the proportion of patients with complete response or partial response
- quality of life (QoL), which is defined as the individual’s perception of their position in life in the context of the culture and value system in which they live in relation to their goals, expectations, standards, and concerns
- adverse events, which are defined as any untoward medical occurrence in a patient or clinical investigation subject who has been administered a pharmaceutical product and which does not necessarily have a causal relationship with this treatment.

### Test medication

The agent for the trial is apatinib. According to the requirements of good clinical practice (GCP), the test medication is uniformly stored, distributed, and recycled by the testing unit. The test drug should be sealed, protected from light, and stored below 25 °C. The validity period is tentatively set for 2 years. Whether other medications can be used during the trial is described in the pharmacy manual.

### Treatment method

**Fig. 1:**
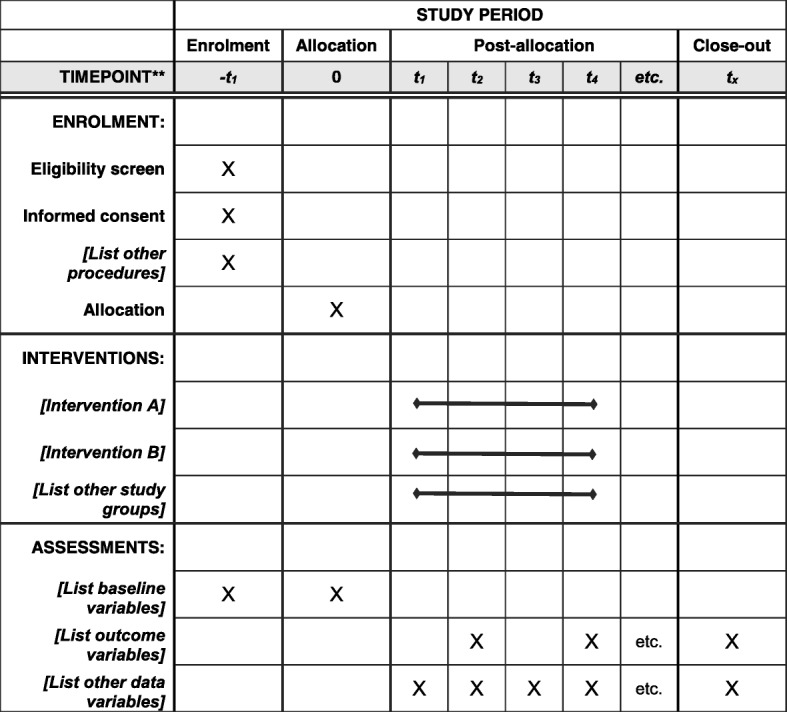
Schedule of enrollment, randomization, and treatment

**Fig. 2:**
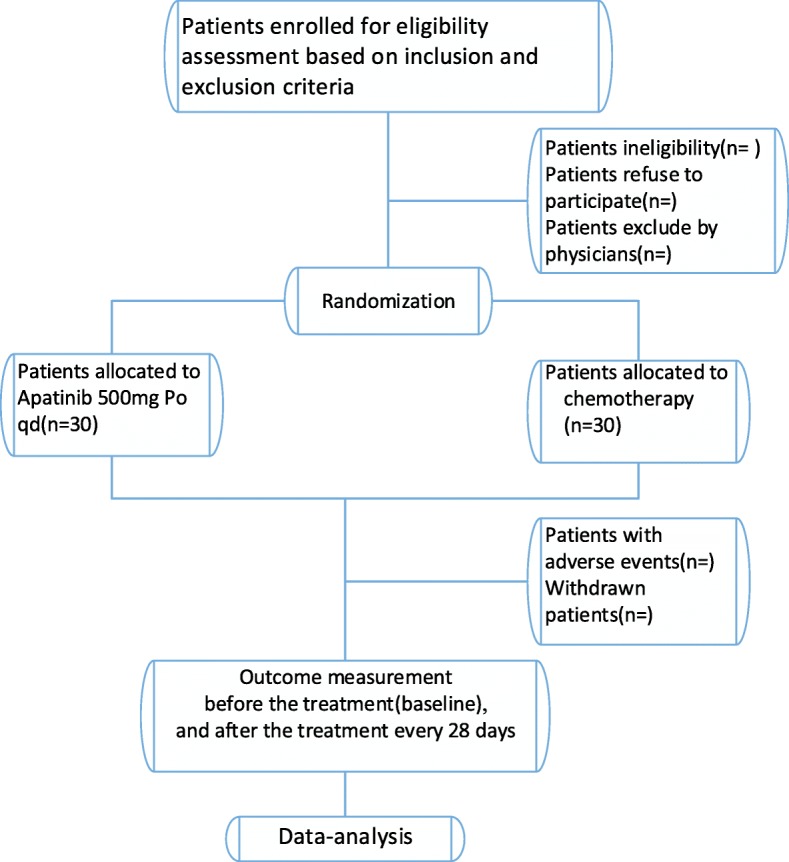
Study flow diagram.

Figure 1 is the study schedule and Fig. 2 depicts the study workflow. The SPIRIT checklist is given in Additional file 1. Enrollment includes a pre-entry screening visit. Table 1 shows the screening checklist. Each participant must sign the protocol-specific informed consent form, which has been approved by the institution review board and follows institutional guidelines. SPSS (version 17) is used to randomly allocate the 60 patients into a treatment group and a control group when they sign the informed consent.

**Table 1:** Checklist for screening visit before enrollment

Item	Yes	No	Note
Informed consent			
Pregnancy test			
Breastfeeding			
Vital signs			
Medical history			
Physical examination			
Bone function examination			
Liver function examination			
Renal function examination			
Coagulation function examination			
Eligibility for the trial			

Each day, participants in the treatment group orally self-administer 500 mg of apatinib (YN968D1) in a 28-day cycle until disease progression or adverse events prohibit further therapy. If they forget to take the medication and remember less than 12 h from the time of the next dose, no agent will be added. Treatment visits will be used to improve and monitor adherence. Table 2 shows the checklist for treatment visits.

**Table 2:** Checklist for treatment visits during the trial

Name:			Date:
Procedures	Yes	No	Note
Medication taken regularly			
Right dose of agent			
Neutropenia			
Thrombocytopenia			
Albuminuria			
Hyperbilirubinemia			
Bleeding			
Hypertension			
Diarrhea			
Hand–foot syndrome			
Fatigue			
Cardiac ischemia			
Gastrointestinal perforations			
Thromboembolic events			
Seizures or convulsions			

This study has an independent data monitoring committee (IDMC), which regularly analyzes the data to assess risk to and benefits for participants. The committee consists of four professional oncologists and one professional statistician, who are independent of the researchers. The IDMC will perform at least one efficacy analysis and one safety analysis during the trial. The efficacy analysis will be conducted when two-thirds of the patients have had an OS event. The safety analysis will be conducted when at least 30 participants have finished one cycle of treatment. Based on the results of the analysis, the IDMC may recommend to the researchers to discontinue the trial or to continue the research after modifying the study plan. They may decide to summarize the data in advance.

Clinical efficacy will be based on a reduction in lesion volumes measured by a computed tomography (CT) or magnetic resonance imaging (MRI) scan, according to RECIST 1.1 [18].

### Dose modification criteria

The Common Terminology Criteria for Adverse Events (version 5.0) will be used for dose modifications and to grade adverse events [19]. The medication will be suspended for any grade 3 non-hematologic toxicity to allow recovery (for less than 2 weeks).

The dose will be modified to 250 mg daily due to toxicity if the following symptoms occur:

(i) grade 3 neutropenia persisting for ≥3 days
(ii) grade 3 thrombocytopenia with bleeding
(iii) grade 3 hypertension previously controlled
(iv) grade 3 or 4 diarrhea
(v) grade 3 hand–foot syndrome
(vi) grade 3 significantly affected normal activity
(vii) grade 3 albuminuria

Apatinib will be discontinued for a participant after any of the following signs of toxicity: grade 4 hypertension, grade 3 albuminuria after two dose modifications, or persistent grade 3 hand–foot syndrome.

### Discontinuation criteria

The discontinuation criteria are as follows:

(i) progression of the disease based on the efficacy evaluation criteria
(ii) in the opinion of the researchers, treatment is not in the best interests of the patient
(iii) unacceptable adverse reactions or serious adverse events
(iv) withdrawal of informed consent
(v) unexpected pregnancy
(vi) death

### Removal from the study

For a participant, the removal criteria are as follows:

(i) misuse or overdose of apatinib
(ii) use of other chemotherapeutics or experimental drug treatments outside the protocol during the trial
(iii) participant included by mistake
(iv) lack of adherence to the therapy for ≥2 weeks

### QoL assessment

QoL and chemotherapy-related toxicity will be assessed before assignment (baseline) and after the first three cycles after study entry. QoL measures include the Edmonton Symptom Assessment Scale (which assesses the symptoms of patients receiving palliative care), the Functional Assessment of Cancer Therapy (Cervix) Trial Outcome Index (which evaluates the therapeutic effect), the Brief Pain Inventory (which measures pain intensity under four different conditions with the intensity of pain being graded from 0 to 10), and the Measure Yourself Concerns and Wellbeing questionnaire (which assesses the concerns and general feeling of wellbeing of patients) [20–23]. These are validated questionnaires.

### Reporting of safety events

For adverse events occurring during the trial, the symptoms, severity, time of occurrence, duration, treatment measures, and outcomes will be recorded. The resident physician will complete a case report form and report to the principal investigator and GCP in a timely manner. Abnormal laboratory test data will be recorded on a case report chart and the test will be repeated at least once a week until recovery or the end of the study.

### Follow-up

All patients will be followed up for 1 year after the study is closed to entry. The efficacy assessments, adverse events, and QoL will be checked every 4 weeks.

### Statistical methods

The null hypothesis (H0) relating to uninteresting levels of activity was determined from an analysis of historical studies whose enrolled patients were expected to behave in a similar way to those eligible for this study [7, 8]. The null hypothesis jointly specifies the probability of a patient experiencing PFS to be less than 10%. If apatinib is not inferior to TP in terms of PFS and is comprehensively superior in terms of other secondary endpoints (such as safety or QoL), apatinib will be the preferred treatment. The corresponding null hypothesis is that the hazard ratio of apatinib to TP is 1.33, the non-inferiority margin. This corresponds to the median survival time of apatinib being inferior to TP (5.34 months) by 1 month, under the proportional hazard assumption [8, 24]. Assuming exponential distributions and that the median survival time of apatinib is 8 months, 27 patients are needed to have 80% power to confirm the non-inferiority with 1-sided α = 5%, after a 1-year follow-up period. Based on considerations such as censored data, the planned sample size is 30. The alternative hypothesis (Ha) is the complement of the parameter space under H0. Secondary endpoints will be OS, objective response rate, disease control rate, and adverse events. Confidence intervals are calculated using the method of Duffy–Santner. PFS is calculated as the time between registration and the earliest time of progressive disease or death. Patients lost to follow-up will be censored at their last disease assessment or at the date they were last known alive, and OS will be calculated as the time between registration and death. The Kaplan–Meier method will be used to estimate PFS and OS, and factors influencing survival will be analyzed using a Cox proportional hazard regression analysis. Statistical analyses will be performed using SPSS (version 17). Treatment-related toxicity will be characterized by frequency and severity according to the organ system affected. Adverse events are based on the maximum toxicity grade for each type of event. Analysis of data is based on the intention-to-treat principal.

### Ethical considerations

This study has been approved by the institutional ethical review board of the Affiliated Hospital of Zunyi Medical College (2017–01). The trial is registered with the Chinese Clinical Trials Registry, number ChiCTR-OIN-17012164. It was registered on 24 July 2017 (http://www.chictr.org.cn/showproj.aspx?proj=20767).

## Discussion

Patients with advanced or recurrent cervical cancer have limited treatment choices and poor prognosis, and chemotherapy is one of the mainstays of treatment. The standard of care for many years has been TP; however, PFS and OS are low. Another chemotherapy option, for instance, targeted treatment, for advanced and recurrent patients is bevacizumab, but this is more toxic. Thus, there is an unmet medical need and a dearth of efficient therapeutic agents.

Apatinib, one of the latest antiangiogenic agents, which is already approved for many solid tumors, has recently been shown to prolong PFS and OS for patients diagnosed with advanced or recurrent cervical cancer. In addition, apatinib is an oral formulation that could improve patients’ QoL. As yet, no prospective trial has focused on apatinib for cervical cancer; therefore, this trial will determine the application, efficacy, and safety of apatinib and will investigate this new type of treatment for advanced or recurrent cervical cancer.

The limitations of our study should also be noted. The gold standard endpoint of randomized clinical trials is OS. In this trial, PFS is recommended as the primary endpoint. Although therapeutics have been approved based on PFS, to what extent PFS can be used as a surrogate for OS in randomized trials of cervical cancer is unknown. Because of the toxicity of apatinib, whether patients will be able to complete the trial is uncertain. Moreover, the small sample size in this clinical trial may lead to an inaccurate analysis.

### Trial status

Recruitment is ongoing.

## Additional file

Additional file 1: SPIRIT 2013 Checklist: Recommended items to address in a clinical trial protocol and related documents. (DOCX 50 kb)

## Abbreviations

CT: Computed tomography
GCP: Good clinical practice
IDMC: Independent data monitoring committee
MRI: Magnetic resonance imaging
OS: Overall survival
PFS: Progression-free survival
PS: Performance status
QoL: Quality of life
RECIST: Response Evaluation Criteria in Solid Tumors
TP: Paclitaxel plus cisplatin
ULN: Upper limit of normal
VEGF: Vascular endothelial growth factor
VEGFR: Vascular endothelial growth factor receptor
